# Body composition, maximal fitness, and submaximal exercise function in people with interstitial lung disease

**DOI:** 10.1186/s12931-025-03195-9

**Published:** 2025-04-02

**Authors:** Owen W. Tomlinson, Anna Duckworth, Laura Markham, Rebecca L. Wollerton, Michael Gibbons, Chris J. Scotton, Craig A. Williams

**Affiliations:** 1 Department of Public Health and Sport Science, Faculty of Health and Life Science, University of Exeter, Heavitree Road, Exeter, EX1 2LU UK; 2 Academic Department of Respiratory Medicine, Royal Devon University Hospitals NHS Foundation Trust, Barrack Road, Exeter, EX2 5DW UK; 3 Department of Clinical and Biomedical Science, Faculty of Health and Life Science, University of Exeter, Heavitree Road, Exeter, EX1 2LU UK; 4 NIHR Exeter Biomedical Research Centre, University of Exeter, Heavitree Road, Exeter, EX1 2LU UK

**Keywords:** Cardiopulmonary fitness, Pulmonary disease, Exercise testing

## Abstract

**Background:**

Cardiopulmonary exercise testing (CPET) is feasible, valid, reliable, and clinically useful in interstitial lung disease (ILD). However, maximal CPET values are often presented relative to body mass, whereas fat-free mass (FFM) may better reflect metabolically active muscle during exercise. Moreover, despite the value of maximal parameters, people with ILD do not always exercise maximally and therefore clinically relevant submaximal parameters must be identified. Therefore, this study assessed peak oxygen uptake (VO_2peak_) relative to FFM, identifying the validity of common scaling techniques; as well as characterising the oxygen uptake efficiency slope (OUES) and plateau (OUEP) as possible submaximal parameters.

**Methods:**

Participants with ILD underwent assessment of body composition and CPET via cycle ergometry during a single study visit. To determined effectiveness of scaling for body size, both body mass and FFM were scaled using ratio-standard (X/Y) and allometric (X/Y^b^) techniques. Pearsons’s correlations determined agreement between OUES, OUEP, and parameters of lung function. Cohens kappa (κ) assessed agreement between OUES, OUEP and VO_2peak_.

**Results:**

A total of 24 participants (7 female; 69.8 ± 7.5 years; 17 with idiopathic pulmonary fibrosis) with ILD completed the study. Maximal exercise parameters did not require allometric scaling, and when scaled to FFM, it was shown that women have a significantly higher VO_2peak_ than men (*p* = 0.044). Results also indicated that OUEP was significantly and positively correlated with DL_CO_ (*r* = 0.719, *p* < 0.001), and held moderate agreement with VO_2peak_ (κ = 0.50, *p* < 0.01).

**Conclusion:**

This study identified that ratio-standard scaling is sufficient in removing residual effects of body size from VO_2peak_, and that VO_2peak_ is higher in women when FFM is considered. Encouragingly, this study also identified OUEP as a possible alternative submaximal marker in people with ILD, and thus warrants further examination.

**Supplementary Information:**

The online version contains supplementary material available at 10.1186/s12931-025-03195-9.

## Introduction

Interstitial lung disease (ILD) describes a large group of pulmonary disorders whereby variable amounts of inflammation and fibrosis result in interstitial and alveolar damage, with resultant decline in lung function [1]. Globally, approximately 2 million people have an ILD [2], accounting for > 120,000 deaths annually [3]. Idiopathic pulmonary fibrosis (IPF) is the most prevalent ILD in the UK, affecting a higher number of males relative to females, with mean age of presentation currently 73.5 years of age [4].

Typically, forced vital capacity (FVC) and diffusion capacity for carbon monoxide (transfer factor; DL_CO_) are utilised for clinical management. However, numerous markers can be utilised to monitor disease progression and evaluate treatment efficacy, with peak oxygen uptake (VO_2peak_) and peak power output (PPO)– the primary outcomes from cardiopulmonary exercise testing (CPET)– having consistently been shown as predictive of poorer clinical outcomes, including mortality and need for transplantation in ILD [5]. Furthermore, weight loss is also predictive of mortality [6], with fat-free mass (FFM) in particular being a significant independent predictor in IPF [7]. Moreover, musculoskeletal strength, notably that of the quadriceps, is reduced in IPF and significantly associated with VO_2peak_ [8].

Given the association between body composition and physical functioning in ILD [9], and their significant effects upon mortality, it is prudent to consider independent and interactive effects of body composition on exercise capacity in ILD. This may be of particular interest when investigating sex-based differences, considering that males appear to have a greater mortality risk than females [10], a risk factor that is reflected when calculating composite scores for disease severity [11, 12]. In addition, females are more likely to have an aerobic fitness below a reported threshold for elevated mortality risk [13], indicating an important interaction between sex and aerobic fitness in ILD.

To date, studies investigating the use of VO_2peak_ in patients with ILD have only controlled for the influencing factor of body mass using traditional ratio-standard scaling techniques [13–15], without verification of the suitability of this process in removing the residual effects of body size from VO_2peak_. It is feasible that use of allometric scaling [16] may be more suitable for controlling for body size– particularly FFM– in this population. Moreover, no studies to date have accounted for the residual effects of body size upon musculoskeletal power (e.g., peak power output, PPO) from CPET, and the subsequent impact upon analyses and interpretation of exercise tolerance in this population.

In addition to these maximal parameters of fitness in people with ILD, it is equally important to consider submaximal parameters in this population. By its nature, CPET is a maximal test, and a number of patients with ILD may not be able to elicit a maximal response, for pathophysiological (e.g., desaturation, cardiac abnormalities) or motivational reasons [17]. The gas exchange threshold (GET) is commonly presented as a submaximal marker from CPET [14, 18], but is often presented as a percentage of VO_2peak_ and therefore paradoxically requires a maximal value against which to anchor. Therefore, measures of aerobic fitness that are not intensity dependent (unlike the GET) are worthy of investigation in this population. Prior work has examined the oxygen uptake efficiency slope (OUES)– a logarithmic transformation of minute ventilation in relation to oxygen uptake– in cardiac [19] and respiratory [20, 21] groups, although not ILD. Moreover, the oxygen uptake efficiency plateau (OUEP)– the highest 90 s average of oxygen uptake relative to ventilation– has also been assessed in cardiac [22] and shows promising discriminative ability in respiratory [23] groups; but has yet to be investigated in ILD.

Therefore, this study sought to characterise: (a) differences in VO_2peak_ and PPO exist between sexes when parameters of body size are fully accounted for via allometric scaling, and (b) submaximal CPET derived parameters in ILD, identifying associations with traditional maximal CPET, and functional spirometry parameters.

## Methods

### Participants

Data for this analysis was obtained from the PETFIB Study (Exploring the potential of Cardio**P**ulmonary **E**xercise **T**esting as a functional marker in patients diagnosed with **FIB**rosing Lung Disease)– aimed at assessing the feasibility of CPET in patients with ILD [17]. This study received ethics approval from the Health Research Authority following review by the South West (Frenchay) Research Ethics Committee (17/SW/0059; IRAS #220189), with all procedures undertaken in line with the Declaration of Helsinki [24].

Recruitment to the study was made via a database screen of eligible participants under the clinical care of the ILD Multi-Disciplinary Team (MDT) at the Royal Devon & Exeter (RD&E) NHS Foundation Trust Hospital. Inclusion criteria included, as noted previously [17]: [1] clinical diagnosis of fibrotic lung disease; [2] age between 40 and 85 years; [3] lung function of FVC > 40% and DL_CO_ >25%; and [4] willing and able to provide informed consent. Moreover, exclusion criteria included: [1] being unable/unwilling to provide informed consent; [2] cardiovascular reasons, including left or right ventricular ejection fraction < 50%; more than mild valvular heart disease; lack of available chest CT images; significant repolarisation abnormalities or arrhythmias identified by resting 12-lead ECG or untoward ECG changes and/or symptoms of ischemia during previous baseline testing of CPET; severe cardiovascular comorbidity or other medical conditions that could contribute to dyspnoea; [3] significant neurological impairment that impairs exercise; [4] pulmonary reasons, including poorly controlled (symptomatic) asthma, or recent exacerbation of asthma (requiring hospitalisation or medical therapy) within the preceding 4 weeks; or forced expiratory volume in 1 s/FVC (FEV_1_/FVC) ratio < 65%; or daytime oxygen therapy; [5] contraindications to exercise testing.

All patients included will have received a clinical diagnosis of fibrotic lung disease as determined by the RD&E MDT, which included a respiratory physician and radiologist, in line with national guidance [25]. Once recruited, all participants provided written and informed consent upon recruitment to the study; whereby a total of 24 participants (17 male, 7 female) with ILD contributed data to this analysis. All anthropometric and pulmonary function data for participants is listed in Table 1.

**Table 1:** Anthropometric and pulmonary function characteristics for 24 individuals with interstitial lung disease

Variable	Whole Group (*n* = 24)	Males (*n* = 17)	Females (*n* = 7)	*P*-value	Effect Size
*Anthropometrics*					
Age (years)	69.8 ± 7.5	70.3 ± 8.3	68.5 ± 5.0	0.59	0.24
Stature (cm)	170.2 ± 7.2	173.0 ± 5.6	163.4 ± 6.5	**0.001**	**1.64**
Body Mass (kg)	80.1 ± 13.4	82.0 ± 13.4	75.3 ± 13.2	0.28	0.56
Body Fat (%)	36.6 ± 10.3	32.3 ± 8.4	47.2 ± 5.9	**< 0.001**	**1.91**
Fat Free Mass (kg)	50.4 ± 10.1	54.9 ± 7.0	39.6 ± 8.0	**< 0.001**	**2.10**
*Pulmonary Function*					
FEV_1_ (L)	2.38 ± 0.54^b^	2.54 ± 0.49^a^	1.96 ± 0.45^a^	**0.021**	**1.21**
FEV_1_ (%_Predicted_)	85.6 ± 16.4^b^	85.6 ± 17.6^a^	85.7 ± 14.4^a^	1.00	0.01
FVC (L)	2.97 ± 0.80^b^	3.19 ± 0.77^a^	2.38 ± 0.58^a^	**0.031**	**1.11**
FVC (%_Predicted_)	83.5 ± 18.7	82.9 ± 19.2	85.1 ± 18.8	0.79	0.12
DL_CO_ (mL.min^− 1^.kPa^− 1^)	4.42 ± 1.00^d^	4.60 ± 1.11^c^	3.97 ± 0.42^b^	0.24	0.64
DL_CO_ (%_Predicted_)	54.1 ± 12.8^b^	54.0 ± 14.8^a^	54.5 ± 5.7^a^	0.94	0.04

All values presented at mean ± standard deviation. ^a^*n*-1, ^b^*n*-2, ^c^*n*-4, ^d^*n*-6 all due to pulmonary function data not being available in medical notes

Statistically significant results, and large effect sizes (> 0.8 (34)) are highlighted in bold

FEV_1_, forced expiratory volume in one second; FVC, forced vital capacity; DL_CO_, diffusing capacity for carbon monoxide

### Physiological measurements

Measures of FEV_1_, FVC and DL_CO_ were retrospectively extracted from pulmonary function test (PFT) data from each participant’s medical records. These data are presented as an absolute value, and as a percent of a predicted value for age, sex, and stature [26, 27], using PFT from the date closest to their CPET. Body fat percentage (and subsequent calculation of FFM) was assessed using air displacement plethysmography (BodPod; COSMED, Rome, Italy).

### Exercise testing

All CPET was undertaken on an electronically braked cycle ergometer (Lode Excalibur; Lode, Groningen, the Netherlands), with participants undertaking an incremental-ramp protocol at a rate of 10 W.min^− 1^. The power at which the participant ceased exercising (through exhaustion or clinical termination) was recorded as PPO. Previous work has shown CPET to be feasible [17], valid and reliable in ILD [28].

Pulmonary gas exchange was recorded using a metabolic cart (Medgraphics Ultima; Medical Graphics UK Ltd., Gloucester, UK), alongside measures of heart rate (HR) via 12-lead electrocardiogram (Welch Allyn CardioPerfect; Hillrom, Chicago, USA), peripheral capillary oxygen saturation (SpO_2_) using a pulse oximeter (Choice MMed MD300C2; ChoiceMMed, Dusseldorf, Germany), and ratings of perceived effort (RPE) and dyspnoea (RPD), on scales of 6–20 and 0–10 respectively [29].

Pulmonary gas exchange data was exported and analysed in 10 s averages, with VO_2peak_ taken as the highest 10-second average and expressed as a percentage of predicted [30]. The GET was determined via the V-slope method [31] and confirmed using ventilatory equivalents for O_2_ and CO_2_. Heart rate reserve (HRR) was calculated by using peak heart rate (HR_peak_) as a percentage of age-predicted maximum (220-Age). Ventilatory limitation was assessed by determining minute ventilation (V_Epeak_) as a percentage of maximal voluntary ventilation (MVV, forced expiratory volume in one-second [FEV_1_] x 40). Tests were deemed to be valid provided they satisfied at least one of the criteria for a maximal effort (e.g. plateau in VO_2_, secondary criteria), as per recent technical standards from the European Respiratory Society [28, 32].

**Fig. 1:**
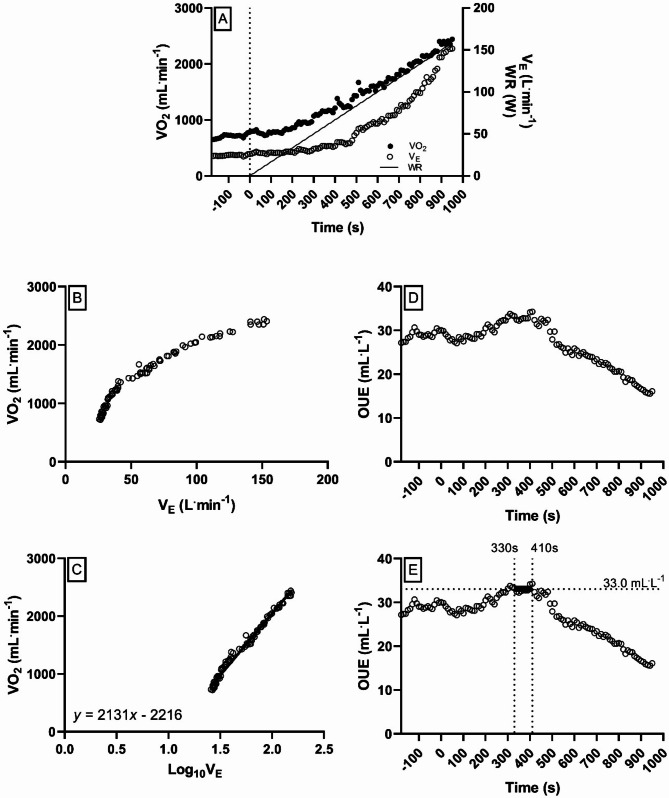
Transformation of VO_2_ and V_E_ data into parameters of OUES and OUEP. **A**: Profile of VO_2_ and V_E_ responses during a cardiopulmonary exercise test with respect to time and work-rate. **B**: Curvilinear ventilatory response to incremental exercise, from the start of the incremental ramp phase to volitional exhaustion (i.e. ˙ VO_2peak_). **C**: The same response profile as (**B**), however ventilation has been log-transformed (base 10). The resultant linear regression in the example above produces a value of 2131 for ‘a’ in equation VO_2_ = a*logV_E_ + b. This value is the oxygen uptake efficiency slope, which is subsequently carried forward for analysis. **D**: Profile of OUE (VO_2_/V_E_) during the during a cardiopulmonary exercise test with respect to time. **E**: Profile of OUE (VO_2_/V_E_) during the during a cardiopulmonary exercise test with respect to time, with the OUEP (highest 90-second VO_2_/V_E_ average) highlighted from 330–410 s; at a value of 33.0 mL.L^−1^. OUE: oxygen uptake efficiency; VO_2_: oxygen uptake; V_E_: minute ventilation; WR: work rate

Derivation of submaximal measures of OUES and OUEP were calculated using previously described methods [23, 33]. For OUES, linear regressions were plotted between VO_2_ and logV_E_, from the start of the incremental phase and up to VO_2peak_, with the slope of this function carried forward as the OUES [33]. For OUEP, the highest 90-second VO_2_/V_E_ average during the course of the CPET was taken forward for analysis; unlike OUES, V_E_ is not transformed in this process [23]. Further, graphical explanation of the derived submaximal measures are provided in Fig. 1.

### Statistical analyses

To determine whether measures of VO_2peak_ and peak power output (PPO) required allometric scaling relative to body mass and FFM, preliminary analyses as described in Supplementary File 1 were undertaken. The necessity of scaling procedures was also assessed for OUES and OUEP.

Independent samples *t*-tests were undertaken to establish differences between sexes for anthropometric and exercise-based variables, including VO_2peak_ and PPO when expressed as an absolute value, and when scaled for body size (using ratio-standard and allometric methods, should both be required). Furthermore, effect sizes, using threshold of Cohen et al. [34], characterised differences between sexes where necessary (small = 0.2, medium = 0.5, large = 0.8).

Correlations between spirometry measures and exercise outcomes were established using Pearsons correlation coefficients, whereby thresholds of Cohen et al. [34]. described the magnitude of coefficients (small = 0.1, medium = 0.3, large = 0.5).

Finally, to assess agreement in classifying patients’ fitness using different parameters, participants were split into equal tertiles of ‘high’, ‘medium’, or ‘low’ fitness for VO_2_ (%_Predicted_), OUES and OUEP in line with previous studies [23]. Agreement between categories was examined via Cohen’s Kappa [35], with strength of this statistic interpreted as ‘poor’ (0.00-0.20) to ‘almost perfect’ (0.81-1.00) [36].

For all null hypothesis significance tests, statistical significance was set at *p* = 0.05 throughout. Statistical tests were undertaken using IBM SPSS Statistics v29 (IBM; Armonk NY, USA).

## Results

Of the 24 participants within this study, the majority (*n* = 17) were diagnosed with IPF, followed by chronic hypersensitivity pneumonitis (*n* = 3), usual interstitial pneumonia (*n* = 2), probable IPF (*n* = 1) and fibrotic organising pneumonia (*n* = 1).

Descriptive data for the whole group, and sexes are presented in Table 1. Stature, body fat percentage, FFM and absolute FEV_1_ and FVC were statistically different between sexes. However, differences in pulmonary function disappeared when normalised to a percentage of predicted. No further anthropometric or pulmonary variable displayed a significant difference between groups. The mean time difference between CPET and PFTs was 7 ± 22 weeks.

### Maximal fitness

As shown in Supplemental File 1, ratio-standard scaling was sufficient in removing the residual effect of body size from VO_2peak_ and PPO in this group, and therefore all data pertaining to VO_2peak_ is expressed in a ratio-standard format herein.

**Fig. 2:**
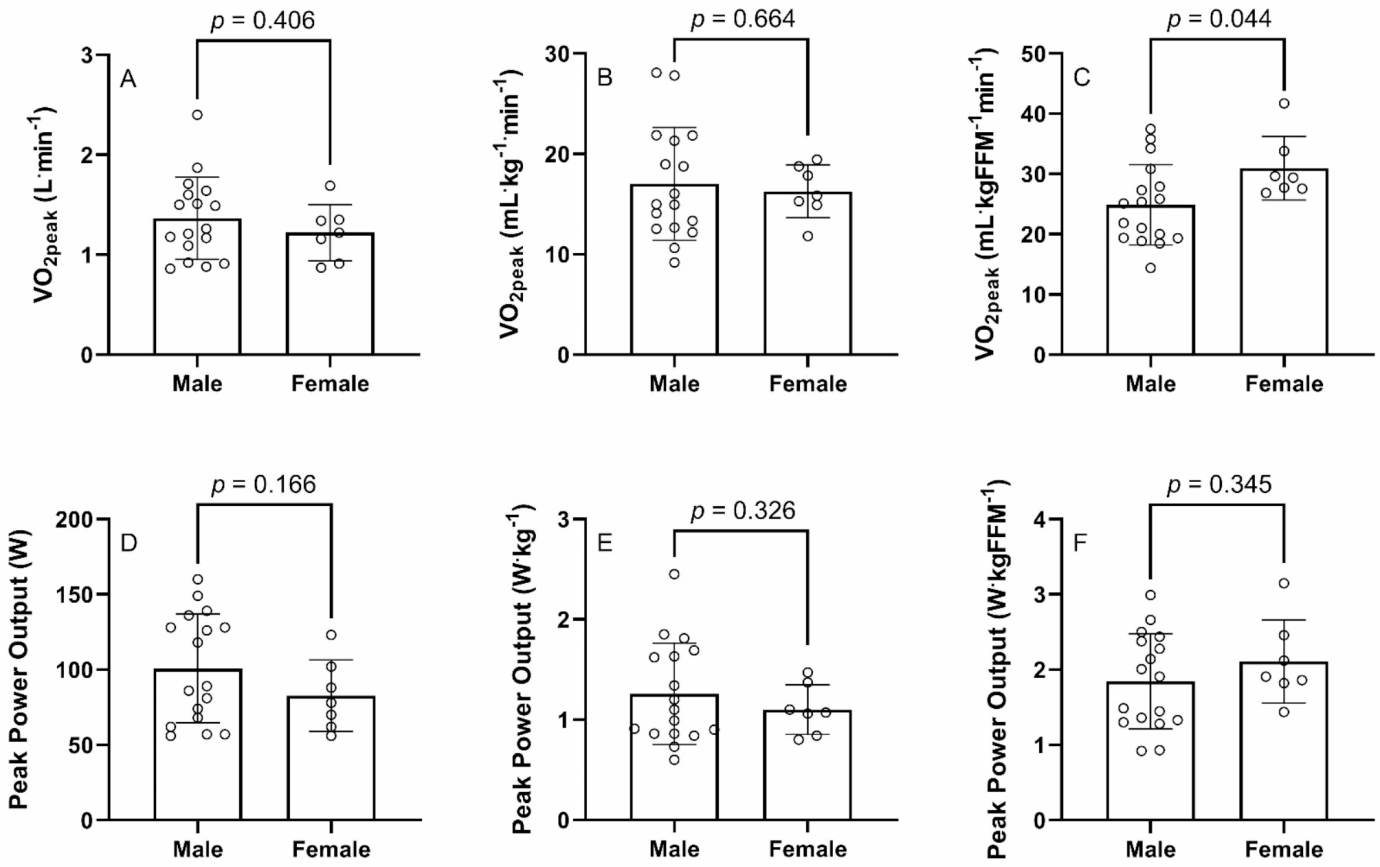
Differences between males and females for VO_2peak_ (**A**-**C**) and peak power output (**D**-**F**), expressed as an absolute value (**A**, **D**), relative to body mass (**B**, **E**), and relative to FFM (**C**, **F**). Scaling in plots B, C, E and F utilise ratio-standard scaling to remove residual effects of body size. FFM, fat-free mass; PPO, peak power output; VO_2peak_, peak oxygen uptake

In comparing VO_2peak_ between sexes, non-significant differences were evident between sexes when expressing VO_2peak_ as an absolute value (M, 1.36 ± 0.41 v F, 1.22 ± 0.28 L.min^− 1^, *ES* = 0.37) and relative to body mass (M, 17.0 ± 5.6 v F, 16.3 ± 2.6 mL.kg^− 1^.min^− 1^, *ES* = 0.16) as shown in Fig. 2 (A, B). However, when expressed relative to FFM, females presented with a significantly higher VO_2peak_ than males (M, 24.9 ± 6.7 v F, 31.0 ± 5.3 mL.kgFFM^− 1^.min^− 1^, *ES* = 0.96), as displayed in Fig. 2C.

In comparing PPO between sexes, non-significant differences were evident between groups when expressed as an absolute value (M, 101 ± 36 v F, 83 ± 24 W, *ES* = 0.54), and relative to body mass (M, 1.26 ± 0.51 v F, 1.10 ± 0.25 W.kg^− 1^, *ES* = 0.35) and FFM (M, 1.85 ± 0.63 v F, 2.11 ± 0.55 W.kgFFM^− 1^, *ES* = 0.43) as shown in Fig. 2 (D-F).

Apart from the scaled VO_2peak_ and PPO data, there were no further differences between sexes for maximal exercise variables (Table 2).

**Table 2:** Exercise based characteristics for 24 individuals with interstitial lung disease

Variable	Whole Group (*n* = 24)	Males (*n* = 17)	Females (*n* = 7)	*P*-value	Effect Size
*Maximal Exercise Parameters*					
VO_2peak_ (L.min^− 1^)	1.32 ± 0.38	1.36 ± 0.41	1.22 ± 0.28	0.41	0.37
PPO (W)	96 ± 34	101 ± 36	83 ± 24	0.17	0.54
VO_2_/PPO (mL.W^− 1^)	14.4 ± 2.6	14.1 ± 1.8	15.0 ± 2.1	0.44	0.48
GET (L.min^− 1^)	0.78 ± 17^a^	0.81 ± 0.18^a^	0.71 ± 0.14	0.25	0.38
GET (%VO_2peak_)	59.7 ± 11.4^a^	59.6 ± 11.3^a^	60.0 ± 12.4	0.93	0.03
V_Epeak_ (L.min^− 1^)	66.5 ± 25.1	71.9 ± 27.1	53.5 ± 13.5	0.10	0.76
V_E_/MVV (%)	71.5 ± 19.6^b^	72.7 ± 22.2^a^	68.4 ± 11.0^a^	0.66	0.22
RER	1.35 ± 0.16	1.36 ± 0.15	1.33 ± 0.22	0.70	0.17
HR_peak_ (beats.min^− 1^)	148 ± 21	144 ± 23	157 ± 12	0.18	0.63
HRR (%_max_)	1.4 ± 13.9	3.5 ± 15.2	-3.8 ± 8.7	0.25	0.53
SpO_2_ (%)	89 ± 5	89 ± 5	89 ± 6	0.96	0.00
RPE	14 ± 2	14 ± 2	14 ± 2	0.94	0.00
RPD	4 ± 1	4 ± 1	4 ± 1	0.82	0.00
*Submaximal Exercise Parameters*					
OUES	1630 ± 333	1622 ± 334	1647 ± 356	0.88	0.07
OUEP (mL.L^− 1^)	27.0 ± 4.9	26.5 ± 5.3	28.4 ± 3.8	0.40	0.38
OUEP (% TTE)	36.0 ± 22.7	41.1 ± 20.2	23.6 ± 25.3	0.09	**0.81**
OUEP (end VO_2,_ L.min^− 1^)*	0.81 ± 0.21	0.85 ± 0.22	0.71 ± 0.12	0.14	0.71
OUEP (end VO_2_, %VO_2peak_)*	63.2 ± 14.4	64.2 ± 14.3	60.7 ± 15.7	0.60	0.24

All values presented at mean ± standard deviation. ^a^*n*-1 due to non-detection of gas exchange threshold; ^b^*n*-2 due to pulmonary function data not being available in medical notes. *As OUEP is a 90-second measure, these values indicate VO_2_ values at the end of this 90-second period

Statistically significant results, and large effect sizes (> 0.8 (34)) are highlighted in bold

GET, gas exchange threshold; HR_peak_, peak heart rate; HRR, heart rate reserve; MVV, maximal voluntary ventilation; OUEP, oxygen uptake efficiency plateau; OUES, oxygen uptake efficiency slope; PPO, peak power output; RER, respiratory exchange ratio; RPD, rating of perceived dyspnoea; RPE, rating of perceived exertion; SpO_2_, saturation of peripheral oxygen; TTE, time to exhaustion; V_Epeak_, peak minute ventilation; VO_2peak_, peak oxygen uptake

### Submaximal fitness

The OUES and the OUEP were not biased by body size, and therefore no scaling procedures have been implemented on these variables.

**Fig. 3:**
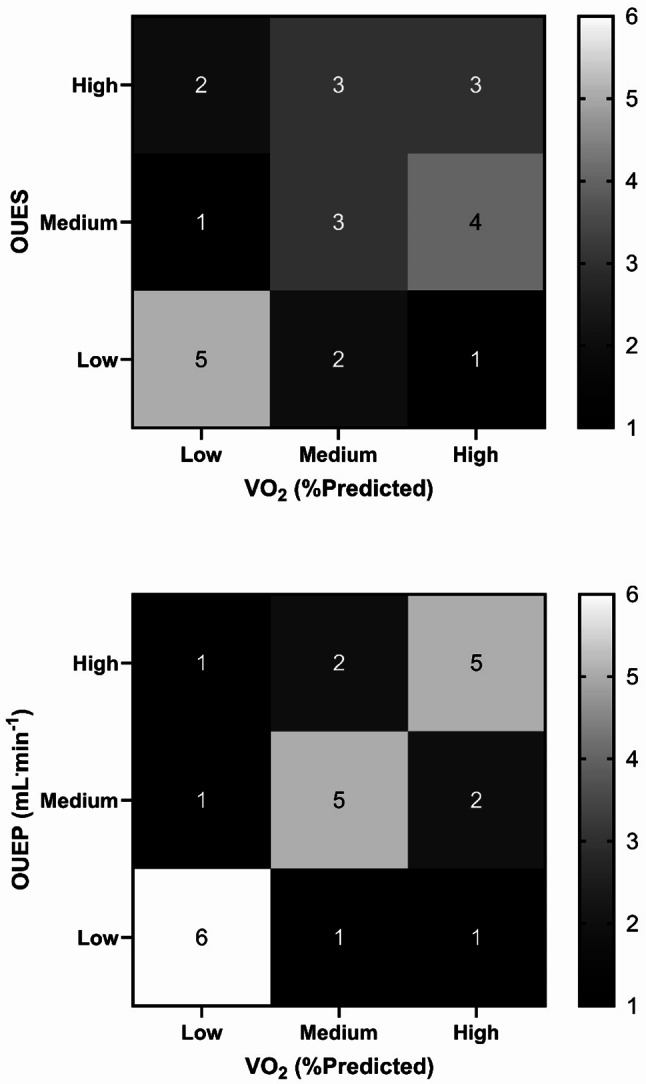
Agreement between VO_2peak_ and OUES (upper plot) and OUEP (lower plot) in number of participants fitting each ‘high’, ‘medium’, and ‘low’ tertiles of fitness. Each box contains number of participants who fit each respective high/medium/low fitness tertile for each measure. A lower number is considered poor, indicating low agreement, whereas a higher number indicates good agreement. OUEP: oxygen uptake efficiency plateau; OUES: oxygen uptake efficiency slope; VO_2_: oxygen uptake

With regards to the OUES, this was not different between sexes (*p* > 0.05; Table 2). It also held large, significant, correlations with DL_CO_, both as an absolute value and as a percentage of predicted (Table 3). When split into tertiles, there was a slight agreement between OUES and VO_2peak_ in their ability to identify high, medium, and low-fitness participants (κ = 0.19, *p* = 0.19; Fig. 3).

**Table 3:** Pearson’s correlation coefficients between parameters of pulmonary function and maximal and submaximal exercise

	VO_2peak_ (L.min^− 1^)	VO_2peak_ (%_Predicted_)	OUES	OUEP (mL.L^− 1^)
FEV_1_ (L)	***r*** **= 0.567** ***p*** **= 0.006**	*r* = -0.072 *p* = 0.751	*r* = 0.072 *p* = 0.750	*r* = 0.314 *p* = 0.155
FEV_1_ (%_Predicted_)	*r* = 0.418 *p* = 0.053	*r* = 0.329 *p* = 0.135	*r* = 0.086 *p* = 0.705	*r* = 0.364 *p* = 0.096
FVC (L)	*r* = 0.456 ***p*** **= 0.033**	*r* = -0.085 *p* = 0.706	*r* = 0.025 *p* = 0.914	*r* = 0.364 *p* = 0.096
FVC (%_Predicted_)	*r* = 0.356 *p* = 0.087	*r* = 0.298 *p* = 0.158	*r* = 0.169 *p* = 0.429	*r* = 0.450 *p* = 0.027
DL_CO_ (mL.min^− 1^.kPa^− 1^)	***r*** **= 0.690** ***p*** **= 0.002**	*r* = 0.153 *p* = 0.546	***r*** **= 0.566** ***p*** **= 0.014**	***r*** **= 0.632** ***p*** **= 0.005**
DL_CO_ (%_Predicted_)	***r*** **= 0.690** ***p*** **< 0.001**	***r*** **= 0.570** ***p*** **= 0.006**	***r*** **= 0.654** ***p*** **< 0.001**	***r*** **= 0.719** ***p*** **< 0.001**

Statistically significant correlations, and large effect sizes (> 0.5 (34)) are highlighted in DL_CO_, diffusion capacity for carbon monoxide; FEV_1_, forced expiratory volume in one second; FVC, forced vital capacity; OUEP, oxygen uptake efficiency plateau; OUES, oxygen uptake efficiency slope; VO_2peak_, peak oxygen uptake

The OUEP was not significantly different between sexes, either as an absolute value, or as an end-VO_2_ value (Table 2). The OUEP as a percentage of time to exhaustion was different between groups, with men taking longer to reach OUEP than women (254 ± 136 vs. 120 ± 133 s). As a whole group, the mean time to reach OUEP was 215 ± 146 s (3 min, 35 s), ranging from 0 to 430 s (occurring during the warm-up; to 7 min, 10 s). The OUEP also held large, significant, correlations with DL_CO_ (larger than OUES; Table 3). When split into tertiles, agreement between VO_2peak_ and OUEP was moderate (κ = 0.50, *p* < 0.01; Fig. 3).

## Discussion

The purpose of this study was to identify the association between parameters of maximal fitness and body composition in those with ILD (predominantly IPF). It was found that ratio-standard scaling is sufficient in removing residual effects of body size, and that females with ILD have a greater VO_2peak_ than males when controlling for body composition. Simultaneously, this study sought to characterise submaximal parameters of fitness in ILD, finding that OUEP holds potential as a functional marker given its similarity to VO_2peak_ in discerning disease severity, and possibly negating the need for patients to exercise to volitional exhaustion.

### Maximal exercise parameters

In characterising parameters of maximal fitness in this group of people with ILD, this analysis has shown that ratio-standard scaling is sufficient in removing residual effects of body size from VO_2peak_ and PPO. Consideration of the explicit nature of body composition in assessing fitness is of utmost importance, given that both predict adverse long term outcomes such as mortality [5, 7], and the prevalence of sarcopenia in people with ILD [37, 38]. Therefore, their independent and interactive effects must be understood to ensure optimal outcomes.

Furthermore, as sex has been shown to have an interaction with VO_2max_ when predicting mortality [13], this is an equally important factor whose interactive effects must be understood. Of further interest, this analysis indicates that when body composition is controlled for, women appear to have a ~ 25% higher cardiopulmonary fitness than male counterparts (Fig. 2C)– an unusual yet novel finding, that warrants replication. Conversely, only one study to date (to the authors’ knowledge) has reported sex differences previously in ILD (thoracic sarcoidosis), showing lower VO_2peak_ as a percent of predicted in women [39], although it is unclear what equations were used and thus comparative interpretation is limited.

Within the present work, as women appear to have higher VO_2peak_, but not higher PPO when FFM is controlled, this may indicate preserved cardiac and/or respiratory function relative to males (who therefore are implied to have a musculoskeletal limitation to exercise tolerance). To further support this, as HR_peak_ is not different between sexes (Table 2), this may indicate a pulmonary limitation to exercise in women, in contrast to men. The functional cause of this pulmonary limitation is unclear given non-significant differences in both FVC and DL_CO_ between sexes in this study (Table 1), but this must also be carefully examined in future studies [40]. Moreover, the possible sex differences in predominant limitations to exercise may influence the personalisation of pulmonary rehabilitation regimens [41].

### Submaximal exercise parameters

In addition to examining maximal parameters, this analysis characterised the OUES and OUEP as possible submaximal surrogates for fitness in ILD. It is noted that OUEP appears the more promising of the two measurements, given a higher correlation with pulmonary function than OUES (Table 3), and a higher agreement than OUES in discriminating tertiles of aerobic fitness (Fig. 3).

One study to date has assessed OUES in ILD [42], noting broadly similar values to the current study, but no insight into its potential clinical application. In contrast to OUES, the current study is the first to examine OUEP in ILD, and appears to have genuine clinical potential. For example, in terms of clinical practice, participants in this study only required a mean of three and a half minutes to obtain a value for OUEP (approximately a third of a CPET duration), a feasible amount of time for patients to exercise. This is in contrast to the OUES, which is typically measured across a full CPET, or an array of points anchored to maximal ability [43]. By having these points anchored to a physiological maximum (such as VO_2peak_), this in turn nullifies the need for a submaximal replacement. As there remains a cohort of patients that will not be able to undergo a full CPET due to clinical contraindications [17], use of the OUEP as an alternative marker of aerobic fitness warrants further investigation.

### Strengths & limitations

The primary strength of this work is the consideration of allometric scaling, relative to FFM; overcoming assumptions to date that both ratio-standard scaling, and use of body mass would be appropriate. Although in the present study, the ratio-standard approach was sufficient in removing residual effects of body size from the present population, this must still be interpreted with caution as it may not be valid for another sample [44], and we encourage the replication of this work across further groups of people with ILD, and its subtypes. In future, all future studies would be prudent to consider allometric scaling as a preliminary analysis for their own respective populations. The limited sample size may also impair interpretation of sex-based differences, but the combined use of null hypothesis significance testing, and effect sizes do aid in overcoming this limitation to an extent.

The use of FFM– a measure that incorporates all non-fat components, including bone, organ tissue, and body water– may not truly reflect skeletal muscle mass (SMM). Instead, a measure such as SMM, or skeletal mass index (SMI), may be a more appropriate parameter against which to scale, as these can be used in the diagnosis of sarcopenia [45], a condition that is present in those with ILDs and is associated with disease severity [37, 38].

However, these parameters were not available via the air displacement plethysmography method, and should be viewed with caution as there is variable agreement between these variables across the rage of prediction equations that are available [46]. Future studies should consider the collection of data specifically related to SMM, as well as FFM, particularly as the latter remains a predictor of survival [7].

In addition, there are some limitations to our sample. Firstly, we did not have a full smoking history for all participants, and therefore we cannot discount any impact of this upon measures of body composition or fitness. Moreover, it must be noted that as the primary aim of this study was to assess feasibility–safety, practicality, acceptability, and demand– of CPET [17], the study was not designed with a sample size calculation a priori to detect observed differences between groups. Utilising differences in VO_2peak_, when scaled relative to FFM (Fig. 2c), observed power (1-β) is deemed to be 0.53, and therefore replication of this work is again called for (comparing two groups of ~ 19 each, if the same power is to be achieved), particularly as so many of the included participants had IPF, and relatively mild disease.

Contrasted against these limitations, this study utilises gold-standard CPET to assess aerobic fitness; a test that is feasible, valid and reliable in this cohort of patients [17, 28]. This ensures that a physiologically maximal response is achieved and interpreted with confidence. Moreover, the aforementioned use of allometric scaling ensures a full interrogation of the association between body size and function is undertaken, further ensuring confident interpretation of data.

## Conclusions

In summary, this study for the first time has verified that ratio standard scaling procedures are sufficient in removing effects of body size from fitness in this set of ILD patients, and in a surprise finding, that women have higher aerobic fitness (relative to body mass) compared to male patients with ILD. Furthermore, the OUEP emerged as a submaximal marker worthy of further investigation in this disease group, particularly for those who may be unable to complete a maximal CPET to volitional exhaustion.

## Electronic supplementary material

Below is the link to the electronic supplementary material.

Supplementary Material 1

## Data Availability

No datasets were generated or analysed during the current study.

## Declarations

### Disclaimer

This project is supported by the National Institute for Health Research (NIHR) Exeter Clinical Research Facility. The views expressed are those of the author(s) and not necessarily those of the NIHR or the Department of Health and Social Care.

## Abbreviations

CPET: Cardiopulmonary exercise testing
DL_CO_: Diffusion capacity for carbon monoxide
FFM: Fat-free mass
FEV_1_: Forced expiratory volume in one second
FVC: Forced vital capacity
GET: Gas exchange threshold
HR: Heart rate
HRR: Heart rate reserve
ILD: Interstitial lung disease
IPF: Idiopathic pulmonary fibrosis
MVV: Maximal voluntary ventilation
OUEP: Oxygen uptake efficiency plateau
OUES: Oxygen uptake efficiency slope
PFT: Pulmonary function test
PPO: Peak power output
RPD: Rating of perceived dyspnoea
RPE: Rating of perceived exertion
SMM: Skeletal muscle mass
SMI: Skeletal mass index
SpO_2_: Peripheral capillary oxygen saturation
V_Epeak_: Peak minute ventilation
VO_2peak_: Peak oxygen uptake
