# Supplementary material for: Body composition, maximal fitness, and submaximal exercise function in people with interstitial lung disease
Source: Respir Res. 2025 Apr 2;26:123. doi: 10.1186/s12931-025-03195-9 (PMC11966908; doi:10.1186/s12931-025-03195-9)
Supplement: Supplementary file 1 — Supplementary Material 1 [file 12931_2025_3195_MOESM1_ESM.docx]

**Supplemental File 1: Methodology & Results of Scaling Procedures**

**METHODS**

Analyses to determine effectiveness of scaling procedures were undertaken in three stages.

1. Pearson’s correlation coefficients established the association between parameters of body mass and FFM, and absolute VO_2peak_ and PPO (X v Y).
2. Ratio-standard scaling was then undertaken by scaling both VO_2peak_ and PPO relative to both body mass and FFM (Y/X), with this ‘ratio-standard’ value subsequently correlated against body mass and FFM (X v Y/X). Should significant correlations exist between corrected VO_2peak_ or PPO and body size, this indicates the residual effects of body size have not been fully removed from VO_2peak_ and PPO, and that allomeric scaling is required.
3. Allometric scaling utilised logarithmic methods detailed previously (1,2). Briefly, the natural logarithm of VO_2peak_ and PPO, and parameters of body size (body mass, FFM) were established, and the regression coefficient (*b*) and associated 95% confidence interval (95% CI) between these parameters identified. Two independent regression analyses were run for each parameter, one whereby sex was controlled for by being entered as a covariate, and one whereby sex was not controlled for. Subsequently, Pearson’s correlations were run between scaled VO_2peak_ and PPO, and body size to determine whether residual effects of body size had been removed (X v Y/X*^b^*).

**RESULTS**

***Correlates with Mass and Fat-Free Mass***

When data was analysed as a whole group, FFM was significantly correlated with absolute VO_2peak_, whereas body mass was not (Figure 1, A-B). Furthermore, PPO was not significantly correlated with either body mass or FFM, (Figure 2, A-B).

When stratified by sex, body mass held a large, non-significant, coefficient with absolute VO_2peak_ in females, but not in males. For FFM, a large, significant, coefficient was observed against VO_2peak_ for females, whereas a medium, non-significant, coefficient was observed for males (Figure 1, E-F). No difference between sexes was observed for the magnitude of the correlation coefficients for VO_2peak_ against body mass (*p* = 0.15) or FFM (*p* = 0.32).

For PPO, no significant correlations were observed between PPO and body mass and FFM, although a large effect size was observed between PPO and body mass for females (Figure 2, E-F). No difference between sexes was observed for the magnitude of the correlation coefficients for PPO against body mass (*p* = 0.16) or FFM (*p* = 0.31).

***Ratio Standard Scaling***

When ratio standard scaling was undertaken, VO_2peak_ was negatively correlated with both body mass and FFM, both to a medium effect, although neither were statistically significant (Figure 1, C-D). When PPO underwent ratio-standard scaling, negative correlations with mass and FFM were found to a small effect, but neither were statistically significant (Figure 2, C-D).

When ratio-standard scaling for body mass was undertaken, negative, non-significant coefficients between scaled VO_2peak_ and body mass were present for both sexes, although a medium effect was observed for females. When scaled for FFM, negative, non-significant, correlations were present for both sexes (Figure 1, G-H). No difference was observed between sexes for the magnitude of the correlation coefficients for VO_2peak_ against body mass (*p* = 0.61) or FFM (*p* = 0.31).

In performing ratio-standard scaling for PPO, a small, negative, non-significant effect was found for males when scaling against body mass, and a negative, non-significant relationship for females, whereas negative, non-significant relationships were found for both sexes when scaling PPO against FFM (Figure 2, G-H). No difference was observed between sexes for the magnitude of the correlation coefficients for PPO against body mass (*p* = 0.76) or FFM (*p* = 0.37).

***Allometric Scaling***

As ratio-standard scaling is sufficient in removing residual effects of body size (body mass and FFM) from whole group, and sex-specific groups, as per Figures 1 and 2, allometric scaling is not a further mandatory requirement for interpretation of data. This is corroborated by the scaling exponents and 95% confidence intervals derived from linear regressions, that span the value of 1.0 for the whole group, and sex-specific groups, when scaling both VO_2peak_ and PPO for body mass and FFM alike (Table 1).

**Table 1.** Scaling exponents for body mass and fat free mass, relative to VO_2peak_ and peak power output.

|  | Body Mass | | | Fat Free Mass | | |
| --- | --- | --- | --- | --- | --- | --- |
|  | *b* | 95% CI | p | *b* | 95% CI | p |
| *VO_2peak_* |  |  |  |  |  |  |
| Whole Group^1^ | 0.421 | -0.254 – 1.095 | 0.209 | 0.577 | 0.075 – 1.079 | 0.026 |
| Whole Group^2^ | 0.383 | -0.325 – 1.091 | 0.274 | 0.896 | 0.181 – 1.611 | 0.017 |
| Male | 0.151 | -0.822 – 1.123 | 0.746 | 0.927 | -0.204 – 2.058 | 0.101 |
| Female | 0.840 | -0.165 – 1.845 | 0.084 | 0.862 | -0.011 – 1.734 | 0.052 |
| *Peak Power Output* |  |  |  |  |  |  |
| Whole Group^1^ | 0.544 | -0.320 – 1.408 | 0.205 | 0.598 | 0.073 – 1.269 | 0.078 |
| Whole Group^2^ | 0.465 | -0.434 – 1.364 | 0.337 | 0.704 | 0.288 – 1.695 | 0.155 |
| Male | 0.263 | -0.980 – 1.506 | 0.659 | 0.718 | -0.822 – 2.259 | 0.336 |
| Female | 0.863 | -0.476 – 2.202 | 0.158 | 0.687 | -0.679 – 2.054 | 0.253 |

^1^No covariates included in model, ^2^Covariate (sex; male/female) included in model. *b* = scaling exponent; 95% CI = 95% confidence interval; p, significance value. VO_2peak_, peak oxygen uptake.


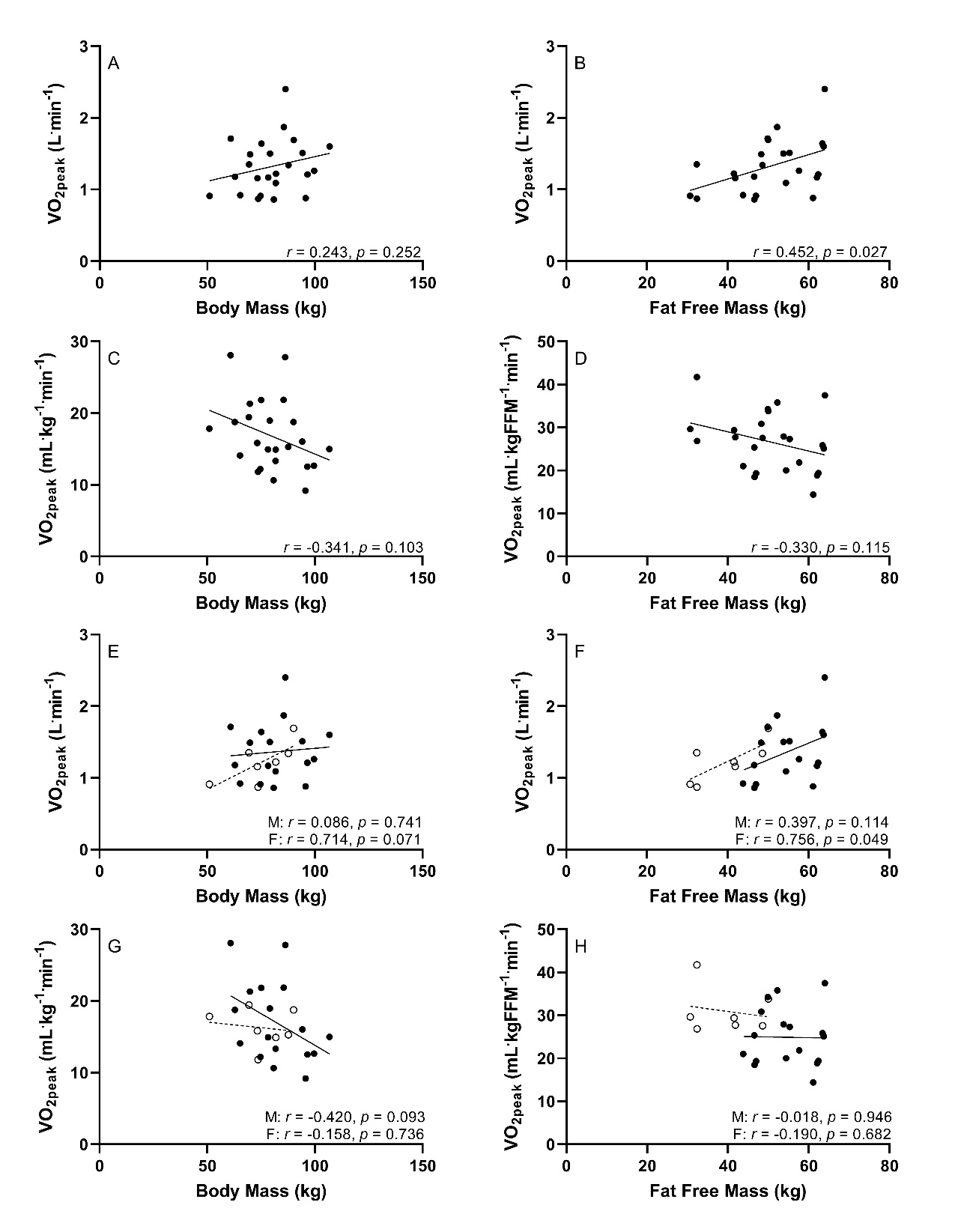


**Figure 1.** A-D: Correlations between body mass and FFM, and VO_2peak_ when expressed as an absolute value (A, B), and relative to both body mass and FFM (C, D), using a ratio-standard method. E-H: Sex-specific correlations between body mass and FFM, and VO_2peak_ when expressed as an absolute value (E, F), and relative to both body mass and FFM, using a ratio-standard method (G, H). Males = black markers with solid line; Females = white markers with dashed line. F, female; FFM, fat-free mass; M, male; VO_2peak_, peak oxygen uptake.


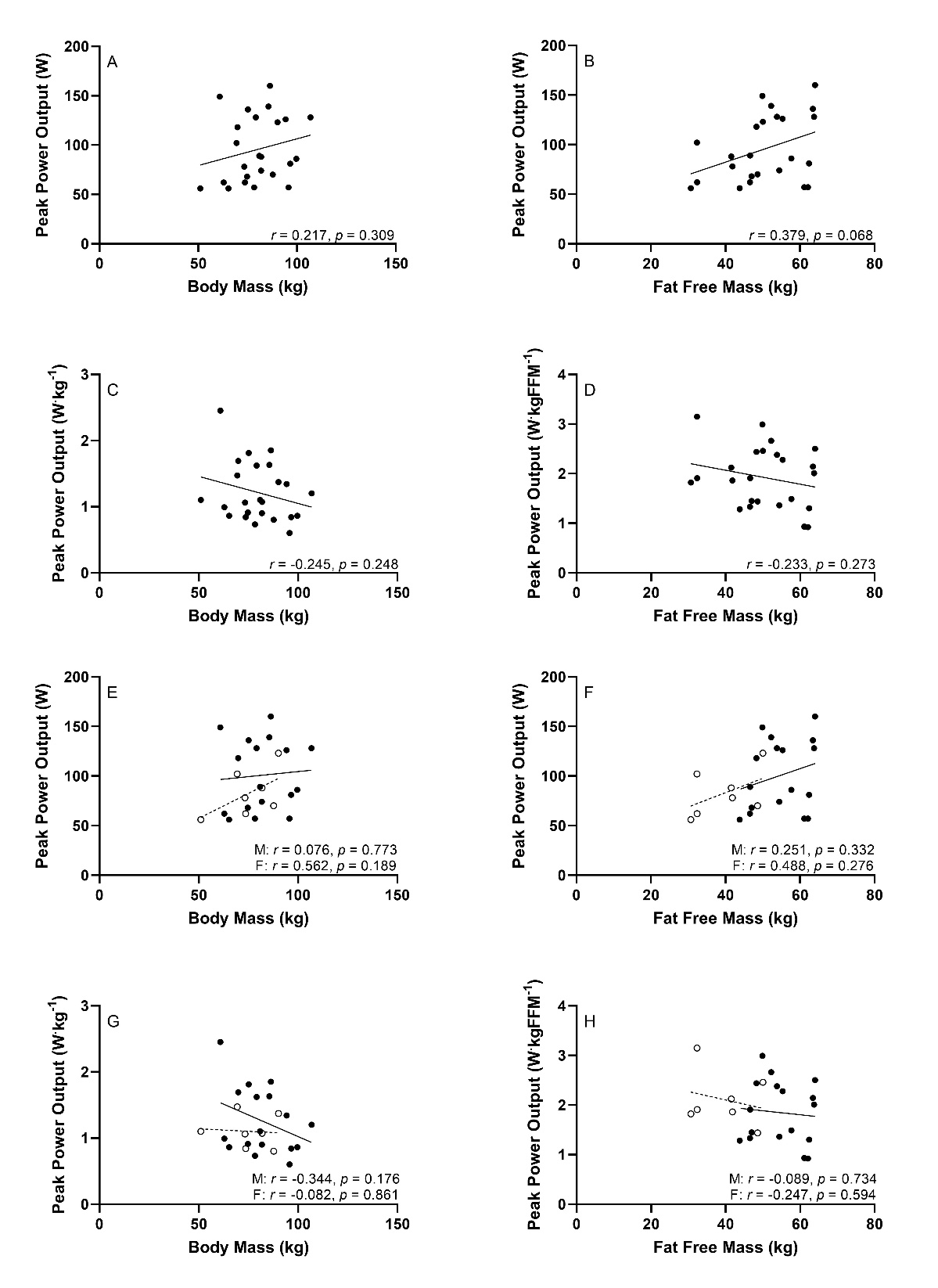


**Figure 2.** A-D: Correlations between body mass and FFM, and peak power output when expressed as an absolute value (A, B), and relative to both body mass and FFM (C, D), using a ratio-standard method. E-H: Sex-specific correlations between body mass and FFM, and peak power output when expressed as an absolute value (E, F), and relative to both body mass and FFM, using a ratio-standard method (G, H). Males = black markers with solid line; Females = white markers with dashed line. F, female; FFM, fat-free mass; M, male; PPO, peak power output.

**REFERENCES**

1. Tolfrey K, Barker A, Thom JM, Morse CI, Narici MV, Batterham AM. Scaling of maximal oxygen uptake by lower leg muscle volume in boys and men. Journal of Applied Physiology. 2006 Jun;100(6):1851–6.

2. Nevill AM, Holder RL. Scaling, normalizing, and per ratio standards: an allometric modeling approach. Journal of Applied Physiology. 1995;79(3):1027–31.
